# Clinical management guidelines for Friedreich ataxia: best practice in rare diseases

**DOI:** 10.1186/s13023-022-02568-3

**Published:** 2022-11-12

**Authors:** Louise A. Corben, Veronica Collins, Sarah Milne, Jennifer Farmer, Ann Musheno, David Lynch, Sub Subramony, Massimo Pandolfo, Jörg B. Schulz, Kim Lin, Martin B. Delatycki, the Clinical Management Guidelines Writing Group, Hamed Akhlaghi, Sanjay I. Bidichandani, Sylvia Boesch, Miriam Cnop, Manuela Corti, Antoine Duquette, Alexandra Durr, Andreas Eigentler, Anton Emmanuel, John M. Flynn, Noushin Chini Foroush, Anne Fournier, Marcondes C. França, Paola Giunti, Ellen W. Goh, Lisa Graf, Marios Hadjivassiliou, Maggie-Lee Huckabee, Mary G. Kearney, Arnulf H. Koeppen, Yenni Lie, Kimberly Y. Lin, Anja Lowit, Caterina Mariotti, Katherine Mathews, Shana E. McCormack, Lisa Montenegro, Thierry Morlet, Gilles Naeije, Jalesh N. Panicker, Michael H. Parkinson, Aarti Patel, Ronald Mark Payne, Susan Perlman, Roger E. Peverill, Francoise Pousset, Hélène Puccio, Myriam Rai, Gary Rance, Kathrin Reetz, Tennille J. Rowland, Phoebe Sansom, Konstantinos Savvatis, Ellika T. Schalling, Ludger Schöls, Barbara Smith, Elisabetta Soragni, Caroline Spencer, Matthis Synofzik, David J. Szmulewicz, Geneieve Tai, Jaclyn Tamaroff, Lauren Treat, Ariane Veilleux Carpentier, Adam P. Vogel, Susan E. Walther, David R. Weber, Neal J. Weisbrod, George Wilmot, Robert B. Wilson, Grace Yoon, Theresa Zesiewicz

**Affiliations:** 1 Bruce Lefroy Centre for Genetic Health Research, Murdoch Children’s Research Institute, Parkville, VIC 3052 Australia; 2 Department of Paediatrics, Melbourne University, Melbourne, VIC Australia; 3 Turner Institute for Brain and Mental Health, Monash University, Clayton, VIC Australia; 4 Monash Health, Clayton, VIC Australia; 5 School of Primary and Allied Health Care, Monash University, Clayton, VIC Australia; 6 Friedreich’s Ataxia Research Alliance, Downingtown, PA USA; 7 Departments of Neurology and Pediatrics, Children’s Hospital of Philadelphia and the University of Pennsylvania, Philadelphia, PA USA; 8 Fixel Center for Neurological Disorders, University of Florida College of Medicine, Gainesville, FL USA; 9 McGill University, Montreal, QC Canada; 10 Department of Neurology, University Hospital, Aachen, Germany; 11 JARA-BRAIN Institute Molecular Neuroscience and Neuroimaging, Forschungszentrum Jülich GmbH and Medical Faculty, RWTH Aachen University, Aachen, Germany; 12 Department of Pediatrics, Children’s Hospital of Philadelphia and the University of Pennsylvania, Philadelphia, PA USA; 13 Victorian Clinical Genetics Services, Parkville, VIC Australia

**Keywords:** Friedreich ataxia, GRADE, Guidelines, Evidence, Recommendations

## Abstract

**Background:**

Individuals with Friedreich ataxia (FRDA) can find it difficult to access specialized clinical care. To facilitate best practice in delivering healthcare for FRDA, clinical management guidelines (CMGs) were developed in 2014. However, the lack of high-certainty evidence and the inadequacy of accepted metrics to measure health status continues to present challenges in FRDA and other rare diseases. To overcome these challenges, the Grading of Recommendations Assessment and Evaluation (GRADE) framework for rare diseases developed by the RARE-Bestpractices Working Group was adopted to update the clinical guidelines for FRDA. This approach incorporates additional strategies to the GRADE framework to support the strength of recommendations, such as review of literature in similar conditions, the systematic collection of expert opinion and patient perceptions, and use of natural history data.

**Methods:**

A panel representing international clinical experts, stakeholders and consumer groups provided oversight to guideline development within the GRADE framework. Invited expert authors generated the Patient, Intervention, Comparison, Outcome (PICO) questions to guide the literature search (2014 to June 2020). Evidence profiles in tandem with feedback from individuals living with FRDA, natural history registry data and expert clinical observations contributed to the final recommendations. Authors also developed best practice statements for clinical care points that were considered self-evident or were not amenable to the GRADE process.

**Results:**

Seventy clinical experts contributed to fifteen topic-specific chapters with clinical recommendations and/or best practice statements. New topics since 2014 include emergency medicine, digital and assistive technologies and a stand-alone section on mental health. Evidence was evaluated according to GRADE criteria and 130 new recommendations and 95 best practice statements were generated.

**Discussion and conclusion:**

Evidence-based CMGs are required to ensure the best clinical care for people with FRDA. Adopting the GRADE rare-disease framework enabled the development of higher quality CMGs for FRDA and allows individual topics to be updated as new evidence emerges. While the primary goal of these guidelines is better outcomes for people living with FRDA, the process of developing the guidelines may also help inform the development of clinical guidelines in other rare diseases.

**Supplementary Information:**

The online version contains supplementary material available at 10.1186/s13023-022-02568-3.

## Introduction

Friedreich ataxia (FRDA) is a multisystem autosomal recessive disease affecting approximately 1 in 29,000 individuals with Caucasian ancestry [1]. Neurological features of FRDA include progressive appendicular and axial ataxia, spasticity, absent lower limb reflexes, dysarthria, visual and hearing dysfunction and impaired vibration sense and proprioception [2]. In addition, non-neurologic features including scoliosis, foot deformity, cardiomyopathy, diabetes mellitus and mental health issues add to the complexity of the disease [3]. The onset of symptoms is on average at 10 years of age but can range from as young as two years to beyond 40 years (in the case of Very Late Onset FRDA) [4]. Individuals with FRDA with an onset of symptoms younger than 15 years lose the capacity to ambulate on average 11.5 years after disease onset [5]. Despite a historical mean age of death reported to be 36 years [6], clinical experience indicates that with targeted care, individuals with FRDA can live for many decades beyond loss of ambulation [7].

Despite significant progress in the search for disease modifying agents, the chronic, progressive course of FRDA cannot yet be significantly slowed. Disease progression continues to have a profound impact on the health and well-being of people with FRDA. Given the rarity of the condition, individuals with FRDA often find it difficult to access experienced specialist multi-disciplinary care for long-term, evidence-based management. Rare diseases present a unique set of challenges in evidence based clinical care. In particular, the lack of adequately powered studies means that many management recommendations are based on low-certainty evidence. Specifically, the developmental and degenerative issues related to FRDA and the need for clinical care across childhood and adulthood requires a diverse approach to clinical management [8]. Recommendations developed without a strong evidence base do not provide clear clinical guidance and run the risk of contributing to sub-optimal care for individuals with rare diseases such as FRDA [9].

In 2014, identifying the requirement for FRDA-specific clinical management guidelines, 39 international expert clinicians wrote the first iteration of the Consensus Clinical Management Guidelines for Friedreich ataxia [10]. In this first iteration, the evidence underscoring the guidelines was evaluated according to the recommendations of the Guidelines International Network (http://www.g-i-n.net/). Recommendations were established according to the criteria developed by the National Health and Medical Research Council (NHMRC) Australia. In the absence of robust evidence, most (62%) of the 142 recommendations in these guidelines were based on the collective expertise of the clinicians and were thus presented as good practice points.

Given the time that had elapsed since 2014, an update of the guidelines was required; however, the challenge of developing guidelines for rare diseases remained. Paucity of available evidence, the predominance of low-quality studies and the inconsistency in metrics used to measure outcomes in FRDA make it challenging to apply standard comparative effectiveness strategies to this and other rare diseases [9]. In particular, the first iteration of the CMGs exemplified the difficulty in generating strong recommendations for diagnosis and/or treatment strategies due to the lack of high-certainty evidence [9].

The time between the first and second iteration of the guidelines provided an opportunity to consider alternative methods of grading the evidence and establishing the strength of the recommendations. The RARE-Bestpractices Working Group has provided a framework for developing guidelines using the Grading of Recommendations Assessment and Evaluation (GRADE) system [11]. Pai and colleagues [9] proposed supplementing the GRADE approach with three strategies specific to rare diseases: (a) reviewing and including literature in like conditions, thus providing “Indirect evidence”, (b) systematic collection of expert observations and/or patient perceptions via “Structured observation forms” and, (c) use of available clinical registry data. This paper describes the application of this approach in developing the updated iteration of clinical management guidelines for FRDA.

## Methods

**Fig. 1:**
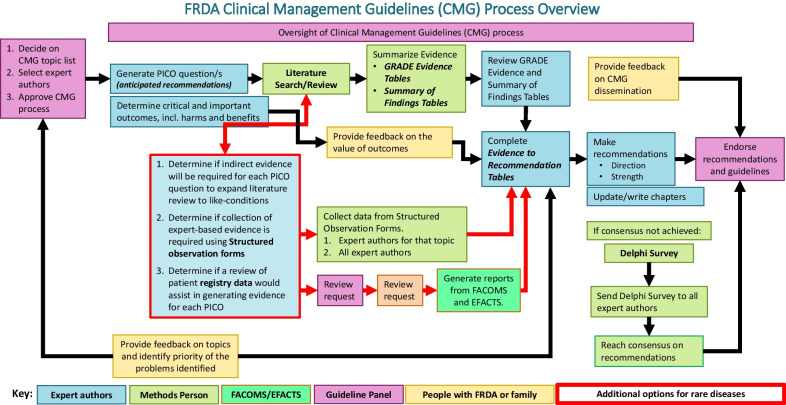
Flow chart of the stages of the guidelines development and stakeholder involvement. Red arrows indicate process for obtaining indirect evidence. CMG = Clinical Management Guidelines; PICO = Patient, Intervention, Comparator, Outcome; FACOMS = Friedreich’s Ataxia Clinical Outcomes Measures; EFACTS = European Friedreich’s Ataxia Consortium for Translational Studies; FRDA = Friedreich ataxia

Figure 1 summarizes the overall process for guidelines development, with each phase described below.

### Stakeholders

The key stakeholders in the development of the guidelines were:

*Guideline panel.* An executive guideline panel comprising seven international clinicians provided oversight of the guideline development process and endorsed the final recommendations.

*Project coordinator*. The panel was supported by a project coordinator and administrative support.

*Expert authors.* International expert clinicians and researchers were invited by the guideline panel to author chapters and develop recommendations for their designated topic.

*Methods experts.* Expert authors were assisted by two methodological experts who completed the literature searches and summarized and synthesized the evidence using the GRADE structure.

*Individuals with FRDA and/or their families.* In contrast to the preceding iteration of the guidelines, individuals with FRDA and/or their families were included throughout the development of the guidelines to provide a lived perspective of FRDA on the development of topics and the final recommendations, in particular the lay summaries of recommendations.

Figure 1 shows how and when these stakeholders contributed to the development of the guidelines.

### Topic list

The guideline panel reviewed the topic list from the previous CMG iteration, identified gaps in the available topics and suggested new topics for inclusion.

### Developing the Patient, Intervention, Comparator, Outcome (PICO) questions

Expert authors were allocated topics relevant to their expertise and were asked to define the scope of their designated topic. Authors were instructed that each recommendation should answer a focused and sensible healthcare question designed according to the Patient, Intervention, Comparator, Outcome (PICO) framework. Individuals with FRDA and/or their families provided feedback on the PICO questions. The PICO questions guided the literature review and generation of other evidence if required.

### Grading of Recommendations Assessment and Evaluation (GRADE)

The GRADE approach is a system for rating the quality of a body of evidence for clinical practice guidelines and grading recommendations in health care. GRADE offers a transparent and structured process for developing and presenting evidence summaries and for carrying out the steps involved in developing recommendations [12]. The GRADEpro Guideline Development Tool (GRADEpro GDT: GRADEpro Guideline Development Tool [Software]; McMaster University and Evidence Prime, 2021; available from https://www.gradepro.org), including the evidence table, summary of findings table and evidence to recommendation table, was used to generate the guidelines. The bespoke structured observation forms used to develop these guidelines were based on those presented in the GRADE tool (see Additional file 1 for an example of a structured observation form).

### Literature search

A literature search for each PICO was completed using a comprehensive search of three electronic databases (Cochrane Library, CINAHL and MEDLINE) for publications from January 2014 to June 2020, including only English-language publications. Reference lists and the authors’ personal libraries were searched for further publications. Randomized, non-randomized controlled and observational studies were included. Papers identified in the 2014 guidelines (published prior to 2014) were included in the review if not superseded by more recent, higher quality literature.

The populations included adults and/or children with FRDA. However, when there was minimal published evidence, populations with like conditions (such as spinocerebellar ataxia and multiple sclerosis) were included in the search.

For each PICO, the review of the literature was summarized and presented to expert authors via the GRADE evidence table and summary of findings table.

### Registry data

In instances where there was limited published evidence on the PICO question, authors had the opportunity to interrogate registry data from two international natural history registries of clinical information from individuals with FRDA: the Friedreich’s Ataxia Clinical Outcomes Measures (FACOMS) and the European Friedreich’s Ataxia Consortium for Translational Studies (EFACTS). The information provided was descriptive only; for example, frequency of use of a medication in individuals with a specific impairment related to FRDA.

### Structured observation forms

If the expert authors anticipated there was limited or no published evidence on the PICO question, structured observation forms were used to systematically document clinical observations by experienced clinicians that could contribute to the strength of the recommendation. In the first instance, the expert authors (clinicians) working on that topic completed the form/s independently. If responses showed large discrepancies, all expert authors were invited to complete the structured observation forms. The questions were targeted at clinicians known to provide healthcare to individuals with FRDA.

### Generation of recommendations

GRADE Evidence to Recommendation tables were used to provide the structure for expert authors to decide the strength of the recommendation and the level of evidence. The evidence base related to each question was evaluated according to the criteria of: problem (is the issue a significant problem?); desirable effects (of the intervention); undesirable effects (of the intervention); certainty of evidence; values; balance of effects; and acceptability (of the intervention). Based on the responses to these criteria, the type (for or against the intervention, or neither) and strength of the recommendation (strong or conditional) were generated. For the rating of the strength of the recommendation, in addition to evidence from studies in FRDA, evidence from like conditions, clinical experience and expert consensus were taken into account when published evidence was not available. In this situation, the level of evidence was rated as “low” or “very low”. Table 1 provides an explanation of the symbols used to grade recommendations.

**Table 1:** Symbols used to denote strength of recommendation and level of evidence

Strength of recommendation	Symbol	Level of evidence	Symbol
Strong for intervention	↑↑	High	⨁⨁⨁⨁
Conditional for intervention	↑	Moderate	⨁⨁⨁◯
Neither intervention nor comparison	–	Low	⨁⨁◯◯
Conditional against intervention	↓	Very low	⨁◯◯◯
Strong against intervention	↓↓		

For strong recommendations, consistent with the GRADE working group we suggested authors should use terminology such as "we recommend…" or "clinicians should…", “clinicians should not…” or “do…”, “don’t…” For conditional recommendations, we suggested less definitive wording, such as "we suggest…" or "clinicians might…" or “we conditionally recommend…” or “we make a qualified recommendation that…”.

Along with each recommendation, authors provided clinical and research justification for the recommendation and a description of subgroups that might need particular consideration in implementing the recommendation, if any.

#### Best practice statements

Best practice statements were included when application of the GRADE framework was not appropriate. This was in circumstances where the reverse would not be credible; for example, ambulant individuals with FRDA who are falling frequently should be offered a detailed assessment of balance and gait. In addition if recommendations from the 2014 guidelines were deemed still relevant, yet not incorporated into a new PICO thus enabling scrutiny from a GRADE framework, they were included as best practice statements.

#### Chapter content

For each topic, expert authors were asked to write a chapter elaborating on the evidence and providing background and context to the best practice statements and recommendations. For chapters that did not include recommendations or best practice statements, expert authors provided an evidence-based overview of the topic.

#### Lay summaries

Expert authors wrote a lay summary of the recommendations related to the topic. Given the target audience for this section is individuals living with FRDA, review of the lay summaries by individuals with FRDA and their families was a crucial component of the guideline development.

#### Achieving consensus

Each of the expert authors needed to agree on the grading of recommendations. In the case of not achieving consensus we proposed the authors use a Delphi survey to achieve consensus.

#### Process of endorsement

The executive panel reviewed the chapter content and recommendations and responded to a set of questions via an online survey (see Additional file 2). If the panel had any reservations about the wording of a recommendation, this was discussed with the relevant authors and agreement was reached. Endorsement was then established after resolution of these issues with the authors.

## Results

Seventy expert clinicians contributed to writing the guidelines. In addition, seven individuals with FRDA and four individuals who care for someone with FRDA provided input from the perspective of the lived experience of FRDA.

Based on the contents of the previous iteration of the guidelines and identified gaps, the guidelines panel defined 17 overall topics for inclusion in the new guidelines, resulting in 17 separate chapters. As shown in Table 2, within the main topics there are specific sub-topics included. For example, there are 11 separate health issues within *Neurological Components of FRDA* (Sects. 3.1 to 3.11). New topics (since 2014) include emergency medicine, digital and assistive technologies and a separate topic (with 3 sub-topics) dedicated to mental health (this was previously included as part of the *Quality of Life* topic). Three of the chapters (1, 2 and 12) provide overviews of topics that did not generate PICOs, and consequently do not contain recommendations, although chapter 12 contains best practice statements. For the other 14 main topics, expert authors used the GRADE rare-diseases process to assess the evidence for 130 PICOs, generating 130 recommendations. Authors were able to achieve consensus on all recommendations without the use of a Delphi survey. In addition to the 130 new recommendations, there are 95 best practice statements included across the 17 chapters.

**Table 2:** Clinical management guidelines for Friedreich ataxia—topics

1	Overview of Friedreich ataxia
2	Potential disease modifying therapies for Friedreich ataxia
3	Neurological components of Friedreich ataxia
3.1	Upper limb dysfunction
3.2	Strength, balance, mobility and reduction of falls in ambulant individuals
3.3	Strength, balance, mobility and reduction of falls in non-ambulant individuals
3.4	Spasticity and spasms
3.5	Dysarthria
3.6	Dysphagia
3.7	Vision
3.8	Lower urinary tract and bowel function
3.9	Sexual function
3.10	Auditory and vestibular function
3.11	Cognitive function
4	The heart and cardiovascular system
5	Surgical and anesthetic considerations
6	Pulmonary function and sleep disturbance
6.1	Reduced pulmonary function and pulmonary infection
6.2	Sleep disordered breathing and hypoventilation
6.3	Restless legs and/or periodic limb movements in sleep
7	Fatigue
8	Pain
9	Orthopedic issues
10	Endocrine and metabolic issues
10.1	Diabetes
10.2	Osteoporosis
10.3	Assessing nutritional status
11	Genetic issues
12	Compound heterozygosity
13	Family planning and pregnancy
14	Mental health issues
14.2	Depression
14.3	Anxiety
14.4	Psychosis
15	Presentations to the emergency department
16	Digital and assistive technologies
17	End of life care
17.1	Advance care planning
17.2	Palliative care
17.3	End of life hospice care

Table 3 lists all best practice statements and recommendations under each topic and sub-topic heading, along with the symbols denoting the strength of the recommendation and the level of evidence for each recommendation (the symbols denoting the strength of the recommendation and level of evidence are explained in Table 1). The full tables of recommendations, each with accompanying justification (based on evidence and clinical expertise) and subgroup considerations, as well as chapter contents and lay summaries are available online. “Evidence to Recommendation” tables showing how each recommendation was developed with summaries of the evidence can also be viewed on the website (https://frdaguidelines.org/).

**Table 3:** Recommendations and best practice statements for each topic

	Strength*	Level of evidence*
3. Neurological components of Friedreich ataxia		
3.1 Upper limb dysfunction		
*Best practice statement*		
At a minimum, annual comprehensive assessment of upper limb function should be conducted by a multidisciplinary team to optimize independence and minimize the effects of primary and secondary symptoms of Friedreich ataxia [16]		
*Recommendations*		
We conditionally recommend intensive upper limb rehabilitation for individuals with Friedreich ataxia in a clinical setting	↑	⨁◯◯◯
We cannot recommend either the use or non-use of sensory specific training of the upper limbs for individuals with Friedreich ataxia in a clinical setting	–	⨁◯◯◯
We conditionally recommend considering upper limb splinting/orthoses for individuals with Friedreich ataxia who experience spasticity, spasm or contracture	↑	⨁◯◯◯
We recommend *against* the use of pharmacological agents (baclofen and botulinum toxin) for specific management of upper limb function in individuals with Friedreich ataxia experiencing spasticity and spasm	↓↓	⨁◯◯◯
We conditionally recommend considering electrical stimulation (but not tDCS or TMS) for management of upper limb function in individuals with Friedreich ataxia in a clinical setting, with appropriate evaluation prior to treatment	↑	⨁◯◯◯
3.2 Strength, balance, mobility and reduction of falls in ambulant individuals with Friedreich ataxia		
*Best practice statements*		
Gait aids should be considered for ambulant people with Friedreich ataxia who are at risk of, or fearful of falling. Individualized assessment is required to ensure the gait aid selected provides the support required to facilitate maximal independence but does not restrict available movement or reduce current balance capacity (due to over-dependence on the gait aid to stabilize)		
Several studies have highlighted the benefits of computer-based or video-based coordination/balance training for individuals with ataxia [17–21]. These papers highlight the importance of prescribing exercise programs that are enjoyable, motivating and sustainable		
Given the significant decline in balance evidence in individuals with Friedreich ataxia, suitably challenging balance exercises should be prescribed in all therapy programs		
*Recommendations*		
For individuals with Friedreich ataxia who are ambulant (with or without an aid), we recommend regular monitoring of ambulation and contributing physical and non-physical factors for mobility decline (such as balance, strength, lower limb spasticity and fear of falling) at least once per year over less regular or informal monitoring	↑↑	⨁◯◯◯
For ambulant people with Friedreich ataxia, we recommend a multi-faceted rehabilitation approach (targeting multiple areas of impairment) over a single focused rehabilitation approach	↑↑	⨁⨁⨁◯
For individuals with Friedreich ataxia who are ambulant, we recommend completing rehabilitation or exercises 3 days per week or more frequently over completing these exercises less than 3 days per week	↑↑	⨁⨁⨁◯
We suggest cardiovascular and endurance exercise training be used over no cardiovascular exercise in ambulant individuals with Friedreich ataxia. Gradual onset and increase in the level of activity, with monitoring for any adverse symptoms, is likely to be a safe approach in those with and without cardiac abnormalities	↑	⨁⨁⨁◯
We suggest lower limb strengthening over no lower limb strengthening in individuals with Friedreich ataxia who are ambulant. In most instances, lower limb strengthening should be one component of a whole exercise or rehabilitation program	↑	⨁◯◯◯
We recommend ankle and foot strengthening and stretching in combination over no strengthening or stretching program in individuals with Friedreich ataxia who are ambulant	↑↑	⨁◯◯◯
In individuals with Friedreich ataxia who are ambulant, we suggest customized orthotics (including lateral ankle support braces, ankle–foot-orthotics) over no orthotic devices when ankle joint kinematics are altered during gait and this has a significant impact on gait. It is important that orthotics are customized and fitted professionally to avoid complications, such as pressure ulcers and incorrectly altered ankle range of motion	↑	⨁⨁◯◯
We cannot recommend either botulinum toxin injection or no pharmacological therapy for ambulant individuals with Friedreich ataxia with ankle or foot spasticity. In selected cases, botulinum toxin injection could be considered after weighing up the potential benefits and harms related to ambulation and dynamic standing balance and therapy-based treatments (such as physiotherapy) have been tried but have not been completely effective. Clinicians should discuss potential negative effects and ensure the individual is aware of the risks prior to this treatment	–	⨁◯◯◯
3.3 Strength, balance, mobility and reduction of falls in non-ambulant individuals with Friedreich ataxia		
*Best practice statements*		
Facilitating ambulant mobility for as long as possible is advantageous to the overall health and well-being of the individual with FRIEDREICH ATAXIA [22]. During and after transition to full-time wheelchair use, it is essential that individuals with Friedreich ataxia maintain the capacity to transfer safely and independently for as long as possible, particularly as length of time since disease onset and time since full-time wheelchair use have been shown to be major indicators for severe deformity requiring intervention [23]		
Core stability, defined as abdominal, gluteal, hip girdle, paraspinal, and other muscles working in concert to provide spinal stability, is imperative for initiation of functional limb movements. Any weakness or reduced motor control of these muscles can impact on a person’s ability to mobilize and balance effectively and independently [24, 25]. People with Friedreich ataxia tend to have weakness in the core stability muscles which then causes problems with balance that can affect their ability to mobilize [26]. Although there is no evidence directly examining core stability in individuals with Friedreich ataxia, numerous studies have found beneficial effects on trunk control and sitting and standing balance in stroke survivors [27, 28]. Therefore, therapy for non-ambulant individuals should include core stability training as one component of their overall program		
Individuals who require a wheelchair for mobility should have these prescribed and customized by a trained clinician. Sensory loss, reduced mobility, scoliosis, diabetes and impaired balance need to be factored into the design and materials chosen to maximize function and minimize falls and pressure area injuries		
*Recommendations*		
For individuals with Friedreich ataxia who are no longer ambulant, we recommend regular monitoring of mobility (including ability to transfer) and contributing physical factors for mobility decline (such as balance, strength, lower limb spasticity and environment set-up) at least once per year over less regular or informal monitoring	↑↑	⨁◯◯◯
For individuals with Friedreich ataxia who are no longer ambulant, we conditionally recommend standing and sitting balance exercises over no balance exercises. Balance exercises should be individually tailored to address each person’s specific impairments and functional goals and minimize risk of falls or fatigue	↑	⨁◯◯◯
We conditionally recommend lower limb strengthening over no lower limb strengthening for individuals with Friedreich ataxia who are no longer ambulant	↑	⨁◯◯◯
We recommend upper limb strengthening versus no upper limb strengthening in individuals with Friedreich ataxia who are no longer ambulant. Caution should be taken to not ‘over-exercise’, especially when there is reliance on the upper limbs to transfer or mobilize	↑↑	⨁◯◯◯
We conditionally recommend cardio-pulmonary exercise over no cardio-pulmonary exercise in individuals with Friedreich ataxia who are no longer ambulant. Gradual onset and increase in the level of activity, with monitoring for any adverse symptoms, is likely to be a safe approach in those with and without cardiac abnormalities	↑	⨁◯◯◯
We conditionally recommend botulinum toxin injections, stretching, and prescription of ankle–foot orthoses for individuals with Friedreich ataxia who are no longer ambulant and who have spasticity and reduced length of the calf muscles. A comprehensive physiotherapy and medical assessment of impairments, mobility status and patient goals should be done to determine the appropriateness of this treatment	↑	⨁◯◯◯
3.4 Spasticity and spasms in Friedreich ataxia		
*Best practice statements*		
People with Friedreich ataxia benefit from assessment for spasticity, pain and spasms (including nocturnal spasms) and incipient or established contracture. This will guide treatment		
Aggravating factors such as infection, pain, constipation, diarrhea, dehydration and pressure sores should be considered and treated in the context of acute onset or exacerbation of spasticity and/or ataxia		
Individuals with Friedreich ataxia, families and caregivers should be educated to monitor the development of spasticity and incipient contractures		
On implementation of an anti-spasticity intervention, individuals with Friedreich ataxia may benefit from reassessment as the treatment of spasticity can unmask weakness and cause deterioration in gait and standing transfers. Individuals should be warned of this phenomenon before anti-spasticity interventions are commenced		
*Recommendations*		
We suggest non-pharmacological (physiotherapy) treatment, such as strengthening and stretching, should be used as a first option in the management of spasticity (and its secondary consequences such as mobility decline), prior to considering pharmacological interventions for individuals with Friedreich ataxia with spasticity. We suggest physiotherapy/rehabilitation interventions (such as strengthening, stretching, serial casting and standing machine stretch) are used to enhance the effects of pharmacological therapy for management of spasticity in individuals with Friedreich ataxia. This should occur when non-pharmacological treatment alone does not address the individual’s problems and/or treatment goals	↑	⨁⨁◯◯
We suggest local pharmacological (i.e. injection of botulinum toxin) management of spasticity for individuals with Friedreich ataxia over no local spasticity management in the following circumstances: a thorough assessment is conducted to weigh up the negative and positive effects of this intervention; spasticity is significantly affecting mobility, function, pain or positioning, and conservative treatment options (such as physiotherapy) are no longer effective	↑	⨁◯◯◯
We conditionally recommend offering systemic pharmacotherapy (baclofen, tizanidine, gabapentin, dantrolene sodium, benzodiazepines, other) for the management of generalized spasticity and spasms in individuals with Friedreich ataxia, with a view to reducing the severity of spasticity and the frequency of spasms and cramps, which may improve mobility and upper limb function and reduce pain	↑	⨁◯◯◯
We cannot recommend either the use or non-use of systemic pharmacotherapy (non-licensed: cannabis, other) to manage spasticity and spasms/cramps in people with Friedreich ataxia	–	⨁◯◯◯
We conditionally recommend *against* the use of neuromodulation for the treatment of spasticity in individuals with Friedreich ataxia	↓	⨁◯◯◯
3.5 Dysarthria in Friedreich ataxia		
*Best practice statement*		
In the absence of strong evidence supporting widespread adoption of treatment for speech problems, interventions to improve the communication skills of listeners (i.e. communication partners of speakers with ataxia) could be incorporated into care plans. These include focused attention during conversations, communicating in quiet environments, and identification of strategies to ameliorate communication breakdowns, with practice of the strategies in a supportive environment		
*Recommendations*		
For people with Friedreich ataxia, we suggest the use of targeted intensive behavioral therapy for improving speech in individuals with dysarthria	↑	⨁⨁◯◯
We cannot recommend either the use or non-use of augmentative and alternative communication (AAC) to treat dysarthria in individuals with Friedreich ataxia	–	⨁◯◯◯
For people with Friedreich ataxia, we suggest that pharmaceutical therapies are *not* used to treat dysarthria	↓	⨁◯◯◯
3.6 Dysphagia in Friedreich ataxia		
*Best practice statement*		
Individuals with Friedreich ataxia who are experiencing difficulties in swallowing should be offered detailed expert counselling on dysphagia management strategies, with a focus on the specificities and mechanisms of dysphagia in degenerative ataxia, as well as their impact on the individual and their friends and families		
*Recommendations*		
For people with Friedreich ataxia, the guidelines panel suggests that behavioral therapies are *not* used as a treatment for dysphagia	↓	⨁◯◯◯
For people with Friedreich ataxia, the guidelines panel suggests employing some compensatory strategies for improving swallowing safety in people with dysphagia	↑	⨁◯◯◯
For people with Friedreich ataxia, we cannot recommend either modified diets/thickened fluids or no diet modification to improve swallow safety	–	⨁◯◯◯
For people with Friedreich ataxia, the guidelines panel suggests neuromuscular electrical stimulation is *not* used as a treatment for dysphagia	↓	⨁◯◯◯
3.7 Vision in Friedreich ataxia		
*Best practice statement*		
Individuals with Friedreich ataxia with vision worse than 20/200 in each eye should be evaluated by a low vision specialist		
*Recommendations*		
We suggest standard treatments for visual impairment that is unrelated to Friedreich ataxia be used as appropriate in individuals with Friedreich ataxia. There is insufficient evidence to recommend the use of medications over conservative therapy for visual impairment unrelated to Friedreich ataxia in individuals with Friedreich ataxia	–	⨁◯◯◯
We suggest standard treatments for optic neuropathy be used as appropriate in individuals with Friedreich ataxia. There is insufficient evidence to recommend the use of medications over conservative therapy for optic neuropathy in individuals with Friedreich ataxia	–	⨁◯◯◯
We suggest standard treatments for optic radiation lesions be used as appropriate in individuals with Friedreich ataxia. There is insufficient evidence to recommend the use of medications over conservative therapy for optic radiation lesions in individuals with Friedreich ataxia	–	⨁◯◯◯
We suggest standard treatments for diabetic retinopathy be used as appropriate in individuals with Friedreich ataxia. There is insufficient evidence to recommend the use of medications over laser treatment for diabetic retinopathy in Friedreich ataxia	–	⨁◯◯◯
3.8 Lower urinary tract and bowel function in Friedreich ataxia		
*Best practice statements*		
Exclusion of a concomitant urinary tract infection and assessment of post micturition residual urine is recommended prior to commencement of treatment [29, 30]		
Antimuscarinic medications may be considered for people with Friedreich ataxia displaying overactive bladder symptoms		
Intradetrusor injections of botulinum toxin A or suprapubic catheterization may be considered as alternative intervention		
In a patient with persistently elevated post void residual volumes in excess of 100 mL, clean intermittent self-catheterization is indicated		
Consider modifying diet and lifestyle to optimize stool consistency and avoid fecal incontinence		
Titrate appropriate laxatives to optimize gut transit, stool consistency and avoid fecal impaction. Consider the use of prokinetic drugs		
Avoid fecal incontinence by treating fecal impaction if present. Facilitate prompt rectal evacuation via use of manual maneuvers, use of suppositories/mini enemas. Consider use of transanal irrigation and biofeedback behavioral therapy		
*Recommendations*		
We conditionally recommend that clinicians enquire about the presence of lower urinary tract (LUT) symptoms when consulting individuals with Friedreich ataxia	↑	⨁◯◯◯
We conditionally recommend that individuals with Friedreich ataxia reporting LUT symptoms have their post-void residual bladder volume measured	↑	⨁◯◯◯
We conditionally recommend consideration of a trial of antimuscarinic agents in individuals with Friedreich ataxia with LUT symptoms reporting urinary storage symptoms	↑	⨁◯◯◯
We conditionally recommend individuals with Friedreich ataxia in urinary retention undergo intermittent catheterization prior to insertion of an indwelling catheter, with suitability for catheterization dependent on their neurological abilities	↑	⨁◯◯◯
We suggest use of non-antibiotic agents, and if unsuccessful, antibiotic prophylactic agents over no antibiotic prophylaxis for the management of recurrent urinary tract infections (UTIs) in individuals with Friedreich ataxia	↑	⨁◯◯◯
We conditionally recommend that clinicians enquire about the presence of bowel symptoms when consulting individuals with Friedreich ataxia	↑	⨁◯◯◯
3.9 Sexual function in Friedreich ataxia		
*Best practice statement*		
Sexual dysfunction in individuals with Friedreich ataxia can have a profound effect on quality of life. Evaluation of sexual function should form part of the routine care of an individual with Friedreich ataxia		
*Recommendations*		
We recommend clinicians enquire about sexual function, including but not limited to erectile or vaginal lubrication dysfunction, the physical capacity to engage in sexual activity and the psychological aspect of the sexual response in sexually active individuals with Friedreich ataxia	↑↑	⨁◯◯◯
We cannot recommend either counseling or no counseling to improve quality of life, self-esteem, management of physical or sensory impairment compromising sexual function, or intimate relationships in sexually active people with Friedreich ataxia reporting sexual dysfunction	–	⨁◯◯◯
We recommend commencing a phosphodiesterase-5 inhibitor in males with Friedreich ataxia who report erectile dysfunction	↑↑	⨁◯◯◯
We recommend *not* commencing a course of natural estrogen in sexually active females with inadequate vaginal lubrication with Friedreich ataxia	↓↓	⨁◯◯◯
We cannot recommend either testing morning serum testosterone or not measuring morning serum testosterone in all sexually active men with Friedreich ataxia reporting sexual dysfunction; however, we suggest morning serum total testosterone should only be tested in males with Friedreich ataxia with clinically suspected hypogonadism, or if first-line treatment of erectile dysfunction is unsuccessful	–	⨁◯◯◯
3.10 Auditory and vestibular function in Friedreich ataxia		
*Best practice statement*		
Assessment of auditory and vestibular function should form part of the standard Friedreich ataxia management protocol. Each individual should be evaluated on an annual basis or more regularly if the individual notices a change in auditory performance or balance		
*Recommendations*		
We recommend that all individuals with Friedreich ataxia undergo auditory and vestibular assessment over not having these assessments	↑↑	⨁⨁◯◯
We cannot recommend either the use or non-use of hearing aids (or cochlear implants) for individuals with Friedreich ataxia with auditory deficits	–	⨁⨁◯◯
We cannot recommend the use of amplification devices over cochlear implants for individuals with Friedreich ataxia with auditory deficits	–	⨁⨁◯◯
We suggest using remote-microphone listening devices over not using any devices for individuals with Friedreich ataxia with auditory deficits	↑	⨁⨁◯◯
3.11 Cognitive function in Friedreich ataxia		
*Recommendations*		
We suggest development and implementation of an educational program for affected individuals and their families and carers to improve information about the potential for, and management of, cognitive dysfunction in individuals with Friedreich ataxia	↑	⨁◯◯◯
We recommend that clinicians should *not* use active neuromodulation (tDCS, TMS) as part of clinical practice to improve cognitive function in individuals with Friedreich ataxia	↓↓	⨁◯◯◯
4. The heart and cardiovascular system in Friedreich ataxia		
4.2 Monitoring		
*Best practice statements*		
An EKG and an echocardiogram should be performed at diagnosis of Friedreich ataxia and then at least annually		
Either 24-h Holter monitoring or Loop monitoring, or possibly both tests, are indicated for individuals with palpitations or other symptoms suggesting the possibility of an arrhythmia. A Loop monitor will be an appropriate additional test when symptoms are infrequent		
Evaluation by a cardiologist should take place if an individual with Friedreich ataxia has cardiac symptoms or abnormal results on cardiac testing		
Patients with Friedreich ataxia being considered for scoliosis surgery, or other major surgery, are at risk of a poor outcome and require a multi-disciplinary approach to the management of heart function during surgery and in the postoperative period		
*Recommendations*		
There is not sufficient evidence to make a recommendation for or against using advanced imaging techniques over standard echocardiography for identifying at-risk individuals with Friedreich ataxia	–	⨁◯◯◯
There is not sufficient evidence to make a recommendation about Holter monitoring for individuals with Friedreich ataxia who do not have symptoms suggesting they might have an arrhythmia	–	⨁◯◯◯
4.3 Arrythmias		
*Best practice statement*		
For treatment of symptomatic arrhythmias in Friedreich ataxia, antiarrhythmic medications (other than betablockers) which are negatively inotropic or are recognized to have a high risk for proarrhythmic effects cannot be assumed to be safe and should rarely, if ever, be used		
*Recommendations*		
We conditionally recommend anticoagulation over no anticoagulation in individuals with Friedreich ataxia with permanent, persistent or paroxysmal atrial fibrillation	↑	⨁◯◯◯
We conditionally recommend attempts to maintain a normal cardiac rhythm over rate control in individuals with Friedreich ataxia and atrial tachyarrhythmias, and also recommend consideration of ablation for those who remain severely symptomatic due to a persistent atrial tachyarrhythmia or frequent paroxysms of an atrial tachyarrhythmia	↑	⨁◯◯◯
4.4 Heart failure		
*Best practice statements*		
There is no therapy with proven cardiac benefits for asymptomatic people with Friedreich ataxia with echocardiographic or cardiac magnetic resonance findings of either a normal heart or increased left ventricular wall thickness but normal ejection fraction		
In adults with Friedreich ataxia and a reduction in left ventricular ejection fraction there is a case for treating according to standard heart failure guidelines		
In individuals with Friedreich ataxia and symptomatic heart failure there is a case for treating according to standard heart failure guidelines		
Women with Friedreich ataxia and a reduction in left ventricular ejection fraction should be advised that pregnancy could result in cardiac decompensation and a greater maternal and fetal risk		
Treatment options such as an ICD and heart transplantation are not contraindicated in Friedreich ataxia, but the appropriateness of such therapy requires careful consideration of the individual’s functional status and their prognosis from non-cardiac morbidities		
*Recommendations*		
We do not suggest using heart failure medication and/or devices for individuals with Friedreich ataxia with a preserved left ventricular ejection fraction	↓	⨁◯◯◯
We conditionally recommend treating individuals with Friedreich ataxia with a reduced left ventricular ejection fraction with medications according to current American Heart Association/American College of Cardiology heart failure guidelines (2013 & 2017 update)	↑	⨁◯◯◯
Advanced heart failure therapies such as a left ventricular assist device, implantable cardioverter-defibrillator, biventricular pacemaker and heart transplantation should be considered for individuals with Friedreich ataxia and heart failure due to a reduced left ventricular ejection fraction, based on consideration of both their cardiac and overall health status	↑	⨁◯◯◯
5. Surgical and anesthetic considerations in Friedreich ataxia		
*Best practice statements*		
Patients with Friedreich ataxia being considered for scoliosis surgery, or other major surgery, are at risk of poor outcomes and require a multi-disciplinary approach to the management of heart function during surgery and in the postoperative period		
As per the 2014 guidelines, it is the view of the expert authors that evaluation by a cardiologist should take place prior to major surgery; cardiac monitoring should take place during major surgery; and major surgery should ideally be conducted in a center with cardiac intensive care facilities		
Careful monitoring of fluid balance is essential in individuals with Friedreich ataxia undergoing stressful events such as scoliosis surgery or hydration therapy in the emergency room setting		
Consideration should be given to appropriate management of peri-operative pain in people with Friedreich ataxia; consideration should be given to the use of nondepolarizing muscle relaxants, in particular accurate assessment of neuromuscular block throughout anesthesia; consideration should be given to avoiding risks associated with hyperkalemia; there should be careful monitoring of fluid balance and cardiovascular function in people with Friedreich ataxia undergoing anesthesia		
Propofol has been used in a large number of individuals with Friedreich ataxia and there is no published evidence that documents adverse events related to the use of propofol in this population. This suggests that theoretical concerns of possible mitochondrial toxicity with propofol are not likely to be clinically meaningful		
*Recommendation*		
We suggest early mobilization following surgery over standard post-operative management for individuals with Friedreich ataxia undergoing surgery. We cannot recommend a particular approach to the timing of getting someone out of bed post-surgery. It is probably best to mobilize as soon as possible with consideration for the following: cardiac/medical status, mobility needs, and decreased reserve of strength	↑	⨁◯◯◯
6. Pulmonary function and sleep disturbance		
6.1 Reduced pulmonary function and pulmonary infection		
*Best practice statement*		
Individuals with advanced Friedreich ataxia should be monitored for reduced pulmonary muscle strength and restrictive lung disease. Based on data from clinical evaluations, pulmonary function testing and possibly sleep studies, interventions such as assisted airway clearance techniques and non-invasive assisted ventilation should be offered		
*Recommendations*		
We conditionally recommend that individuals with advanced Friedreich ataxia be monitored* at least annually for restrictive lung disease and sleep disordered breathing (SDB) *Monitoring should include a respiratory symptom check list (dyspnea, orthopnea, episodes of apnea during night, poor sleep, morning headache, decreased concentration and attention, fatigue, treated chest infection within the past few months), a sleepiness questionnaire and a fatigue scale. Annual (or more frequent) pulmonary function testing should be performed to include forced vital capacity (FVC), maximum inspiratory pressure (MIP) and maximum expiratory pressure (MEP), peak expiratory cough flow (PECF), SpO_2_ and partial pressure of end tidal CO_2_ (PetCO_2_)	↑	⨁◯◯◯
We conditionally recommend *against* monitoring for restrictive lung disease and sleep disordered breathing at diagnosis of Friedreich ataxia rather than at later stages of the disease, as there is no evidence that this would be of benefit	↓	⨁◯◯◯
In individuals with Friedreich ataxia and impaired airway clearance (PECF < 270 L/min or FVC < 50% predicted), we suggest assisted coughing (mechanical/manual) be implemented to assist in airway clearance and reduce the prevalence of chest infections	↑	⨁◯◯◯
In people with respiratory weakness and restrictive lung disease with Friedreich ataxia, we conditionally recommend chest physiotherapy to improve respiratory function, reduce prevalence of chest infection, reduce dyspnea and improve airway clearance function	↑	⨁◯◯◯
We conditionally recommend non-invasive assisted ventilation for patients with Friedreich ataxia and documented restrictive lung disease meeting the following thresholds: FVC < 50% predicted; maximum inspiratory pressure < 60 cm H_2_O; nocturnal hypercarbia (pCO_2_ > 50 mm Hg for ≥ 2% of sleep time or a 10 mm Hg increase in pCO_2_ compared to awake baseline pCO_2_ for ≥ 2% of sleep time); nocturnal hypoxia (SpO_2_ ≤ 88% for ≥ 2% of sleep time or 5 min continuously); or apnea–hypopnea index ≥ 5. Daytime hypoventilation indicated by hypercarbia of > 45 mm Hg or baseline PO_2_ < 95% on room air is also an indication for nocturnal assisted ventilation	↑	⨁◯◯◯
We cannot recommend either respiratory strength training or no respiratory strength training for people with Friedreich ataxia and respiratory weakness and restrictive lung disease. We suggest that in selected patients with respiratory weakness, supervised respiratory training be considered with monitoring of respiratory parameters and for adverse effects such as exhaustion	–	⨁◯◯◯
6.2 Sleep disordered breathing and nocturnal hypoventilation		
*Best practice statement*		
Individuals with advanced Friedreich ataxia should be monitored for sleep disordered breathing using clinical questionnaires and assessments. When obstructive sleep apnea is diagnosed, appropriate night-time ventilatory assistance or an appropriate alternative treatment should be offered		
*Recommendations*		
We conditionally recommend that individuals with advanced Friedreich ataxia be monitored* at least annually for restrictive lung disease and sleep disordered breathing (SDB) *Monitoring should include a respiratory symptom check list (dyspnea, orthopnea, episodes of apnea during night, poor sleep, morning headache, decreased concentration and attention, fatigue, treated chest infection within the past few months), a sleepiness questionnaire and a fatigue scale. Annual (or more frequent) pulmonary function testing should be performed to include forced vital capacity (FVC), maximum inspiratory pressure (MIP) and maximum expiratory pressure (MEP), peak expiratory cough flow (PECF), SpO_2_ and partial pressure of end tidal CO_2_ (PetCO_2_)	↑	⨁◯◯◯
We conditionally recommend *against* monitoring for restrictive lung disease and sleep disordered breathing at diagnosis of Friedreich ataxia rather than at later stages of the disease, as there is no evidence that this would be of benefit	↓	⨁◯◯◯
In individuals with Friedreich ataxia and sleep disordered breathing/sleep apnea and/or evidence of nocturnal hypoventilation, we suggest non-invasive ventilation be implemented to assist in fatigue; sleepiness; quality of night time sleep; blood gas parameters; and cardiac function	↑	⨁◯◯◯
6.3 Restless legs and/or periodic limb movements in sleep		
*Recommendations*		
We conditionally recommend the use of prevention strategies/lifestyle changes (such as reduction of alcohol and nicotine use) over no prevention strategies/lifestyle changes or medication in individuals with Friedreich ataxia with restless legs syndrome	↑	⨁◯◯◯
We conditionally recommend investigating serum ferritin levels in individuals with Friedreich ataxia presenting with symptoms of restless legs syndrome over not checking ferritin. Serum ferritin is usually measured in combination with serum iron and transferrin saturation. Given that serum ferritin can be raised when inflammation is present, acute and chronic inflammation should be assessed at the same time by doing a white cell count and measuring C-reactive protein (CRP)	↑	⨁⨁◯◯
We suggest alternative/complementary treatments should *not* be used over no treatment/medication/lifestyle/ physiotherapy for restless legs syndrome in Friedreich ataxia	↓	⨁◯◯◯
We suggest iron supplementation could be trialed for treatment of RLS in individuals with Friedreich ataxia and serum ferritin < 50 mcg/ml, but only If other treatments have been tried and are not effective. Clinicians should only consider a trial of iron supplements if serum ferritin is < 50 mcg/ml and no acute or chronic inflammation is present, with close monitoring and a review to assess any adverse effects on ataxia after 3 to 6 months. If an individual has RLS and serum ferritin > 75 mcg/ml, they should not be given iron supplements	↑	⨁◯◯◯
We conditionally recommend medication for individuals with Friedreich ataxia with RLS which interferes with sleep (with or without associated PLMS) over no medication. Gabapentin and pregabalin are the preferred choice of pharmacological treatment of RLS in Friedreich ataxia as they are as effective as levodopa but do not have the same side-effects. The dopamine agonists pramipexole and ropinirole may be helpful but should be used with caution in Friedreich ataxia due to the risk of augmentation of RLS symptoms. If PLMS is present it should be treated if the individual has disabling symptoms. Levodopa may be used intermittently when disabling RLS/PLMS symptoms are present since augmentation of RLS occurs only with long-term use. Given that levodopa alleviates symptoms of RLS rapidly, a 'test dose' of levodopa may be used to confirm a diagnosis of RLS in an individual with Friedreich ataxia	↑	⨁◯◯◯
7. Fatigue in Friedreich ataxia		
*Best practice statement*		
Sometimes it may be difficult for individuals with Friedreich ataxia to identify if they suffer from fatigue. In this case, they should be offered a detailed assessment of fatigue including administration of a standardized assessment such as the Modified Fatigue Impact Scale [31] and enquiry about the impact of fatigue on the capacity to participate in daily activities		
*Recommendations*		
We suggest behavioral management (mindfulness, energy conservation, use of assistive devices, sleep hygiene, stress reduction, cognitive behavioral therapy, relaxation, avoiding multitasking) may assist in managing fatigue in individuals with Friedreich ataxia	↑	⨁⨁◯◯
We suggest a physical activity program be used to manage fatigue in individuals with Friedreich ataxia	↑	⨁⨁⨁◯
We suggest some pharmacological intervention (antioxidants, antidepressants) may have value in managing fatigue related to Friedreich ataxia	↑	⨁⨁◯◯
We cannot recommend either alternative therapies/physical modalities (i.e., light therapy, cooling therapy, pulsed electromagnetic devices, acupuncture) or no alternative therapies/physical modalities be used for all individuals who report fatigue with Friedreich ataxia	–	⨁◯◯◯
We cannot recommend any specific diet over usual diet to assist in managing fatigue in individuals with Friedreich ataxia	–	⨁◯◯◯
8. Pain in Friedreich ataxia		
8.2 Neuropathic pain		
*Best practice statements*		
Neuropathic pain may be treated with gabapentin, pregabalin, lamotrigine, amitriptyline or duloxetine		
A detailed sensory assessment and examination by a clinician familiar with the peripheral neuropathy related to Friedreich ataxia will establish the extent of neuropathy		
Protective foot care is important		
Preventative measures such as review of daily activities, transfers and wheelchair positioning may reduce the incidence of focal neuropathies		
*Recommendations*		
We suggest the use of oral medication over no medication in individuals with Friedreich ataxia who have painful neuropathy	↑	⨁⨁◯◯
We suggest that clinicians should *not* consider the use of oral supplements to manage neuropathic pain in individuals with Friedreich ataxia	↓	⨁◯◯◯
We suggest the use of topical agents over no treatment in the management of neuropathic pain in Friedreich ataxia	↑	⨁◯◯◯
8.3 Pain not related to neuropathy		
*Recommendations*		
We conditionally recommend the use of oral medication over no medication to manage pain in individuals with Friedreich ataxia	↑	⨁◯◯◯
We conditionally recommend the use of physical therapy in the first instance, over no physical therapy or medication to manage pain in individuals with Friedreich ataxia. If physical therapy is not effective, as per the previous recommendation we conditionally recommend the use of oral medication over no medication	↑	⨁◯◯◯
We conditionally recommend that injections for musculoskeletal pain can be tried in individuals with Friedreich ataxia when medications are not tolerated or are insufficiently effective	↑	⨁◯◯◯
We cannot recommend the use of implanted devices (spinal stimulator or pain pump) over oral medication for pain in individuals with Friedreich ataxia. Clinicians may consider implanted devices in individuals with severe symptoms in whom less invasive treatments have proven insufficiently effective	–	⨁◯◯◯
9. Orthopedic issues in Friedreich ataxia		
9.2 Scoliosis		
*Best practice statements*		
Spinal examination must be part of the multidisciplinary approach for individuals with Friedreich ataxia and should be performed regularly		
Individuals with Friedreich ataxia with a spinal curve between 20° and 40° and/or between the ages of 10 and 16 years should be observed for curve progression		
Bracing may not reduce or stop the progression of curves; however, it may be valuable in delaying surgical correction in the young child		
People with Friedreich ataxia with a scoliosis > 40° may be considered appropriate for surgical correction		
Consideration should be given to delaying surgical intervention in ambulant individuals with Friedreich ataxia		
All people with Friedreich ataxia considered for scoliosis surgery require extensive pre-operative evaluation and planning regarding cardiac and pulmonary function		
*Recommendations*		
We suggest surgery should *not* be used over conservative therapy for individuals with scoliosis and Friedreich ataxia. Expert opinion suggests that conservative therapy, including the use of bracing during the time of rapid growth in children/adolescents (age 10 to 16 years), may assist in avoiding or delaying surgery	↓	⨁◯◯◯
We recommend that age should be considered when contemplating scoliosis surgery for individuals with Friedreich ataxia	↑↑	⨁◯◯◯
We recommend stratifying individuals with Friedreich ataxia according to cardiac status when considering scoliosis surgery	↑↑	⨁◯◯◯
9.3 Foot deformity		
*Recommendations*		
We conditionally recommend orthopedic surgery for individuals with foot issues with Friedreich ataxia if it is considered such surgery would assist in foot alignment to facilitate mobility and/or safe and effective transfers	↑	⨁◯◯◯
We conditionally recommend the use of orthotics (including ankle–foot orthotics, ankle braces and in-shoe orthotics) for individuals with foot issues with Friedreich ataxia	↑	⨁◯◯◯
We cannot recommend either physiotherapy (including stretching, soft-tissue work) or no physiotherapy for individuals with foot issues with Friedreich ataxia	–	⨁◯◯◯
10. Endocrine and metabolic issues in Friedreich ataxia		
10.1 Diabetes mellitus		
*Best practice statements*		
All individuals with Friedreich ataxia should have annual screening for diabetes mellitus and symptoms of hyperglycemia (polyuria, polydipsia, unexplained weight loss) should be reviewed with patients and families		
Management of diabetes mellitus in Friedreich ataxia should involve diabetes specialists and take an individualized approach		
*Recommendations*		
We recommend *at least* annual screening for diabetes mellitus with HbA1c and fasting plasma glucose in children and adults with Friedreich ataxia, with consideration of an oral glucose tolerance test if impaired fasting glucose or pre-diabetes (from HbA1c) is identified, over universal screening with an oral glucose tolerance test. The decision to pursue intermittent oral glucose tolerance tests should be discussed with patients and families on an individualized basis	↑↑	⨁⨁◯◯
There is insufficient evidence to favor either insulin alone or metformin or novel glucose-lowering therapies (e.g., SGLT2i, GLP1RA, DPPIVi) as initial therapy for adults with lower-risk diabetes mellitus (HbA1c < 8.5%, no ketones, no acute hyperglycemia) with Friedreich ataxia. We suggest an individualized approach with either insulin alone, and/or a glucose-lowering agent, with the choice of medication patient dependent, particularly because of the heterogeneity in Friedreich ataxia-related diabetes mellitus	–	⨁◯◯◯
There is insufficient evidence to favor either insulin alone or insulin in combination with metformin or novel glucose-lowering therapy (e.g., SGLT2i, GLP1RA, DPPIVi), in adults with Friedreich ataxia and higher-risk diabetes mellitus (HbA1c ≥ 8.5%, ketones, or acute hyperglycemia). Insulin is an appropriate treatment but possible risks and benefits of adding other medications are unknown in Friedreich ataxia and treatments must be individualized	–	⨁◯◯◯
We suggest using insulin alone rather than insulin and other glucose-lowering therapy as the primary treatment for most children (under 18 years old) with Friedreich ataxia-related diabetes mellitus	↑	⨁◯◯◯
10.2 Osteoporosis		
*Best practice statements*		
Addressing identified nutritional deficiencies in calcium and vitamin D in individuals with low bone health is considered best practice in both adults (e.g., National Osteoporosis Foundation guidelines: https://www.bonesource.org/clinical-guidelines) and children [32]. However, there may be risks (e.g., hypercalciuria) with universal supplementation. There are no data to suggest any additional benefit of calcium or vitamin D supplementation for bone health beyond the standard daily recommended intakes		
Individuals with Friedreich ataxia and osteoporosis should be managed by clinicians with relevant experience		
*Recommendations*		
We recommend universal screening assessment of bone mineral density (DXA scan, fracture history) over risk-stratified screening in adults with Friedreich ataxia, given the availability of anti-osteoporosis medications that have been shown to prevent pathological fractures due to low bone mineral density (osteopenia, osteoporosis) in related populations	↑↑	⨁⨁◯◯
Clinicians should consider universal screening of children with Friedreich ataxia for low bone density via DXA; at minimum, a risk-stratified approach is recommended	↑	⨁◯◯◯
We conditionally recommend annual screening for vitamin D deficiency over no screening or risk-stratified screening in individuals with Friedreich ataxia	↑	⨁⨁◯◯
We conditionally recommend *against* routine calcium and vitamin D supplementation for individuals with Friedreich ataxia, but vitamin D and calcium supplementation should be considered for those with identified nutritional and/or biochemical deficiencies in calcium and vitamin D intake as these are known risk factors for decreased bone health and may contribute to longer-term fracture risk	↓	⨁⨁◯◯
We conditionally recommend anti-resorptive (bisphosphonate) therapy for children with Friedreich ataxia who may not yet have an aBMD Z-score of -2.0 or lower, but have at least one clinically significant fragility fracture. We recommend that treatment be undertaken by a clinician with relevant expertise, such as a pediatric endocrinologist	↑	⨁◯◯◯
10.3 Assessing nutritional status		
*Best practice statements*		
All individuals with Friedreich ataxia should have height, weight, and BMI measured at least annually. In the minority of individuals who cannot safely stand with assistance, an alternate measurement could be used (e.g., ulnar length, supine length, seated height, or arm span)		
The United States Preventive Services Task Force (USPSTF) and other organizations recommend routine screening for nutritional status with BMI (in children, adolescents, and adults)		
*Recommendations*		
We suggest using standard BMI thresholds to define underweight and overweight in children and adults with Friedreich ataxia	↑	⨁◯◯◯
11. Genetic issues in Friedreich ataxia		
11.2 Testing adult siblings		
*Best practice statements*		
Requests for carrier testing by at-risk adult siblings are best managed on a case-by-case basis; there is no evidence to support the routine provision or refusal of carrier testing for Friedreich ataxia		
All at-risk siblings identified as having Friedreich ataxia pre-symptomatically and their families would benefit from immediate post-test counseling and psychosocial support and referral for appropriate neurological and cardiac surveillance		
*Recommendations*		
We suggest that adult siblings of a person with Friedreich ataxia, who do not wish to have genetic testing to confirm whether or not they have Friedreich ataxia, be offered echocardiography to see if they have any cardiac signs that may require treatment	↑	⨁◯◯◯
We suggest that adult siblings of people with Friedreich ataxia should be offered a physical examination. They should be made aware that this could identify signs of Friedreich ataxia. Absence of signs of Friedreich ataxia does not mean that they will not be found to have biallelic pathogenic variants in *FXN*. The older the individual with a normal examination, the less likely they are to have biallelic pathogenic variants in *FXN*	↑	⨁◯◯◯
11.3 Testing minor siblings		
*Recommendations*		
If an asymptomatic at-risk minor sibling of a person with Friedreich ataxia has not had genetic testing to confirm whether or not they have the genetic predisposition to Friedreich ataxia, we suggest they should be offered echocardiography to assess if they have cardiac morbidity that may require treatment. The minor (when of maturity to understand) and their parents should be made aware that echocardiography can identify that the child has Friedreich ataxia on the basis of the presence of typical cardiac findings. They should also be made aware that a normal echocardiogram does not exclude the diagnosis of Friedreich ataxia	↑	⨁◯◯◯
We suggest minors at risk of Friedreich ataxia (siblings of people with Friedreich ataxia) should be offered psychological support to assist with dealing with anxiety that may arise from being at risk of developing the condition	↑	⨁◯◯◯
We cannot recommend the routine offer of pre-symptomatic genetic testing over refusal to offer testing for immature minors at risk of Friedreich ataxia. Each situation is unique and should be managed on a case-by-case basis with referral to a team with expertise in pre-symptomatic genetic testing and the related issues	–	⨁◯◯◯
We conditionally recommend testing over refusal of testing for an asymptomatic mature at-risk minor who requests genetic testing for Friedreich ataxia. When a mature minor requests testing, a referral should be made to a team with expertise in pre-symptomatic genetic testing for Friedreich ataxia and the related issues	↑	⨁◯◯◯
11.4 Testing other relatives		
*Best practice statement*		
Carrier testing should be first undertaken on the closest relative as a negative result means that genetic testing of more distant relatives may not be necessary		
11.5 Provision of information on GAA repeat size		
*Recommendation*		
Although not all testing laboratories report *FXN* GAA repeat sizes, we suggest that when repeat sizes are reported for pre-symptomatic testing for Friedreich ataxia and the individual is homozygous for *FXN* GAA expansions, this information is provided to the tested individual upon request. Where GAA repeat sizes are provided to the tested individual we suggest that the individual is informed that there is a negative correlation between GAA1 size and age at onset, but the range of age of onset for any GAA1 size is broad and the age of onset for that person cannot be predicted with certainty	↑	⨁◯◯◯
11.6 Optimal genetic support services		
*Best practice statements*		
Referral to a clinical geneticist or genetic counselor should be considered on diagnosis of Friedreich ataxia		
All individuals identified pre-symptomatically and their families would benefit from immediate post-test counseling and psychosocial support and referral for appropriate neurological and cardiac surveillance		
There is no evidence to support routine use of any pharmacological therapies in patients diagnosed with Friedreich ataxia pre-symptomatically		
12. Friedreich ataxia due to compound heterozygosity		
*Best practice statements*		
If a person compound heterozygous for a FXN GAA expansion and a point mutation/deletion has a similar phenotype to those with Friedreich ataxia due to homozygosity for GAA expansions, they should be managed as per the guidelines in this document		
If spastic ataxia is the predominant phenotype, then the main management issue is that of spasticity and the guidelines for management of spasticity should be followed		
It should never be assumed that other features of typical Friedreich ataxia (e.g., cardiomyopathy, diabetes) will not be present in individuals with compound heterozygosity; therefore, monitoring for these should take place		
13. Family planning and pregnancy in Friedreich ataxia		
*Best practice statements*		
Testing for carrier status of reproductive partners should be made available to couples where one member has Friedreich ataxia, prior to conception in order to advise the couple of the risk of having a child with Friedreich ataxia and to offer appropriate counseling		
When possible, it is advisable for women to have children earlier in their disease course		
Pregnancy in women with Friedreich ataxia and a reduced left ventricular ejection fraction and/or a history of heart failure is likely to be associated with an increased risk of adverse maternal and fetal outcomes. Pre-pregnancy counseling for such women is suggested, including consultation with a multidisciplinary team that should include a cardiologist and an obstetrician		
Women with Friedreich ataxia should be encouraged to proceed with pregnancy if they wish to do so and if their cardiac status is adequate [33]		
Glucose tolerance testing should be performed between 24 and 28 weeks of gestation or earlier for individuals deemed to be at high risk by their practitioner [34]		
Women with Friedreich ataxia should have close monitoring by a cardiologist during pregnancy		
There is insufficient evidence to determine if magnesium sulfate can be safely administered to women with Friedreich ataxia with preeclampsia		
There is insufficient evidence to determine if common beta-agonist tocolytic agents can be safely administered to women with Friedreich ataxia experiencing pre-term labor		
Pregnant women with Friedreich ataxia and deep venous thrombosis should be treated with heparin as opposed to warfarin [35]		
Vaginal delivery can be expected for most pregnancies in women with Friedreich ataxia [36]		
Close fetal monitoring during delivery is recommended [37]		
If cesarean section is medically indicated, epidural or spinal anesthesia can generally be safely used in women with Friedreich ataxia [38, 39]		
*Recommendation*		
We conditionally recommend that women with Friedreich ataxia with reduced ejection fraction with or without heart failure be advised of the risks of mortality and morbidity associated with commencing or proceeding with a pregnancy	↑	⨁◯◯◯
14. Mental health issues in Friedreich ataxia		
14.2 Depression		
*Best practice statements*		
Individuals with Friedreich ataxia require regular evaluation in terms of risks for developing depression and/or other mental health issues		
Individuals with Friedreich ataxia A may benefit from regular counseling to assist in adjusting to transitional events and possibly prevent the emergence of related depression		
Individuals with Friedreich ataxia identified with depression should be treated with established interventions including counseling ± pharmacological agents		
The risk of suicide in individuals with Friedreich ataxia should be considered and managed proactively		
*Recommendations*		
We conditionally recommend the use of antidepressant medication in individuals with Friedreich ataxia who present with symptoms of depression	↑	⨁◯◯◯
We recommend counseling or therapy over no counseling in individuals with Friedreich ataxia who present with symptoms of depression	↑↑	⨁◯◯◯
We recommend lifestyle changes (exercise, diet, social activities) either prior to or in conjunction with other interventions, including antidepressants, for individuals with Friedreich ataxia who have symptoms of depression	↑↑	⨁◯◯◯
14.3 Anxiety		
*Recommendations*		
We conditionally recommend the use of anti-anxiety medication in individuals with Friedreich ataxia who present with symptoms of anxiety	↑	⨁◯◯◯
We recommend counseling or therapy over no counseling for individuals with Friedreich ataxia who present with symptoms of anxiety	↑↑	⨁◯◯◯
We conditionally recommend *against* lifestyle changes as a primary intervention to treat anxiety in individuals with Friedreich ataxia, favoring anti-anxiety medication or counseling prior to or in conjunction with any lifestyle changes	↓	⨁◯◯◯
14.4 Psychosis		
*Recommendations*		
We conditionally recommend the use of antipsychotic medication in individuals with Friedreich ataxia with confirmed episodes of psychosis	↑	⨁◯◯◯
We conditionally recommend *against* the use of counseling or therapy over antipsychotic medication in cases of acute psychosis in Friedreich ataxia	↓	⨁◯◯◯
15. Emergency presentations in Friedreich ataxia		
15.1 Chest pain		
*Recommendation*		
We recommend that chest pain assessment in Friedreich ataxia should incorporate the following considerations: coronary artery disease is not more or less common than in the non-Friedreich ataxia population; ECG is usually abnormal in Friedreich ataxia in the absence of coronary disease; troponin can be elevated in patients with Friedreich ataxia for a non-coronary reason and should not be assumed to indicate an acute coronary syndrome; the possibility of increased incidence of pulmonary embolism due to the sedentary nature of the disease. If troponin is elevated, then serial troponin assessment is very important. In the setting of left ventricular hypertrophy an episode of atrial fibrillation can lead to angina-like pain	↑↑	⨁⨁◯◯
15.2 Other emergency presentations		
15.2.1 Musculoskeletal presentations		
*Best practice statements*		
Pain may exacerbate spasticity/spasms, and therefore pharmacological management of spasticity may be required to minimize the impact on physical function and mobility		
Treatment of any orthopedic injury may require a coordinated multi-disciplinary approach in an inpatient rather than outpatient or community setting, due to the potential for decline in function. This should be assessed on an individualized basis		
*Recommendations*		
We suggest thorough and careful multi-disciplinary assessment of the causes and effects of falls should be performed for individuals with Friedreich ataxia presenting to the emergency department with a fall over standard assessment, taking into consideration factors such as neurological progression, cardiac arrhythmia, hypotension, uncontrolled or newly presenting diabetes. More advanced imaging such as CT or MRI scan may be necessary to assess for any spinal cord or nerve root compression, or for complications related to any prior scoliosis surgery, such as rod infection or migration	↑	⨁◯◯◯
We suggest that trauma management approaches that minimize the time spent immobilized might be considered, with careful consideration of the risks and benefits related to each individual with Friedreich ataxia	↑	⨁◯◯◯
15.2.2 Urinary tract infection		
*Recommendation*		
The assessment of UTIs in the emergency department for individuals with Friedreich ataxia should not be fundamentally different from standard care in individuals without Friedreich ataxia. However, we recommend reviewing self-catheterization technique, if appropriate, and performing diagnostic evaluation to assess for elevated residual urine (including a bladder scan) and morphological causes (including an ultrasound of the kidneys, ureters and bladder (KUB) and outpatient cystoscopy)	↑↑	⨁◯◯◯
15.2.3 Diabetes emergency		
*Recommendation*		
We suggest routine diabetes screening using an appropriate test in all individuals with Friedreich ataxia who present to the emergency department, even in the event of a seemingly unrelated complaint. Once hyperglycemia has been determined, management in individuals with Friedreich ataxia should not be fundamentally different to the management of hyperglycemia in individuals without Friedreich ataxia, but with the following consideration: individuals with Friedreich ataxia and diabetic ketoacidosis may need a higher dose of insulin therapy as a result of insulin deficiency and insulin resistance in Friedreich ataxia	↑	⨁◯◯◯
16. Digital and assistive technologies		
*Best practice statements*		
Comprehensive assessment to identify barriers to independence should be conducted by a multidisciplinary team to identify appropriate customizable digital and assistive technology to optimize independence and occupational participation and performance, and to enhance quality of life		
Compensatory or remedial interventions with digital and assistive technology may improve independence for individuals with Friedreich ataxia		
Prescription of a manual or powered wheelchair or scooter should be preceded by an assessment of the home/school/work and community environment the equipment will be used in		
A comprehensive prescription of a manual or powered wheelchair or scooter should be completed by a qualified clinician familiar with the specific issues related to Friedreich ataxia		
A validated assessment and evaluation tool for wheelchair and seating prescription may be used to guide the process of prescription and evaluation		
In prescribing a manual wheelchair and seating system, functional capacity should not be impeded for the sake of an anatomically correct seated posture		
Appropriate training should be provided for the safe use of a wheelchair or scooter in the home or community environment		
Suitability of the seating and wheelchair system should be evaluated on an annual basis in adults and biannually in children		
*Recommendations*		
For individuals with Friedreich ataxia with impaired independent gait/mobility, we suggest the use of customized assistive technology for mobility (e.g., modified vehicle, wheelchair, transfer devices, walkers, and adaptive seating and positioning) to enhance independence in mobility, quality of life, and social and occupational participation, and to reduce falls	↑	⨁◯◯◯
For individuals with Friedreich ataxia with impaired upper limb functionality, we suggest the use of customized assistive technology for personal care and environmental control (e.g., iPad, home apps, smart watches, Alexa/Siri) to enhance independence in daily activities, quality of life, and social and occupational participation	↑	⨁◯◯◯
For individuals with Friedreich ataxia and fatigue, a risk of falls, poor sleep, diabetes and/or cardiomyopathy, we suggest the use of health monitoring/alert devices (e.g., monitoring heart rate, steps, sleep, ECG, activity, healthy eating, medication, glucose for Friedreich ataxia-related diabetes) to enhance independence in daily activities and quality of life, and improve medication control	↑	⨁◯◯◯
For individuals with Friedreich ataxia with impaired communication and workplace difficulties, we suggest the use of customized assistive technology for communication and the workplace (e.g., writing, speech, computer use, tablets, workplace design [adaptive seating and positioning], vision and hearing) to enhance independence in communication, improve quality of life and increase occupational participation	↑	⨁◯◯◯
17. End-of-life care		
17.1 Advance care planning		
*Best practice statement*		
Given the advances in medical technology that can prolong life in the setting of advanced disease, all adults with Friedreich ataxia should consider appointing a designated healthcare representative. This person may be a trusted family member, close friend or independent advocate who will act on the authority of the person with Friedreich ataxia. It is important that the representative understands the care the person with Friedreich ataxia would like or not wish to have. Treatment preferences can be set out in an advance care plan in case the person with Friedreich ataxia becomes disabled or unable to communicate their own wishes		
*Recommendation*		
We conditionally recommend advance care planning (ACP) for individuals with Friedreich ataxia who have reached adulthood, have major complications such as heart failure, have experienced a significant change in their mobility, have dysphagia or have barriers to communication, bearing in mind that the published literature on ACP is in heart failure. ACP should also address the "future loss of dignity" by putting in place a safeguard that a person’s own values and wishes be respected in their care. This would help to implement a degree of control over a disease which is often out of the control of the person with Friedreich ataxia	↑	⨁⨁◯◯
17.2 Palliative care		
*Best practice statement*		
All patients with Friedreich ataxia should be offered palliative care at specific time-points, such as with their transition to adult care, as well as when clinical milestones occur (e.g., loss of ambulation, onset of dysphagia, and with development of symptomatic heart failure) so that quality of life and future care preferences can be discussed		
*Recommendation*		
We suggest that a palliative care consultation should be considered for individuals with Friedreich ataxia complicated by heart failure, particularly when there is a large burden of symptoms, poor perceived quality of life, or an upcoming medical decision to be made about which there is uncertainty of benefit or a significant impact on quality of life	↑	⨁⨁◯◯
17.3 End-of-life hospice care		
*Best practice statement*		
Individuals with Friedreich ataxia should receive end-of-life care tailored to their individual healthcare-related values, with a focus on alleviating burdensome symptoms and promoting their opportunity to choose the location of care if such a preference exists		
*Recommendation*		
We suggest that people with Friedreich ataxia with advanced heart failure would benefit from hospice support when their goals align with a comfort-focused approach and the individual’s prognosis meets eligibility criteria—usually a life expectancy of 6 months or less if the disease runs its natural course	↑	⨁◯◯◯

*Symbols denote the strength of the recommendation and the level of supporting evidence according to GRADE (see Table 1 for definitions of symbols)

Table 3 shows that the majority of recommendations are supported by a very low or low level of evidence, reflecting the lack of high-quality clinical studies of many aspects of FRDA. However, in many cases, a conditional or strong recommendation was made based on the criteria specified in the GRADE rare-disease process. For 23 of the 130 PICO questions, the authors were not able to generate a recommendation either for or against the intervention. In these cases, the authors considered there was not enough data in FRDA or other conditions, or enough solid clinical experience to make any judgement about whether these particular interventions may be useful or not in FRDA. Sixteen recommendations were either conditional or strong recommendations *against* the intervention, while the rest (n = 91) were conditional or strong recommendations *for* the intervention.

## Discussion

FRDA presents with a unique, heterogeneous phenotype that spans both childhood and adulthood. As such, CMGs for FRDA need to provide expedient clinical guidance for clinicians, particularly those less familiar with the condition. The diverse manifestation and complexity of FRDA mandates appropriate CMGs developed in a transparent manner, via a process able to synthesize available evidence and delivered in a clear and concise manner [9]. Consistent with the approach taken by Pai and colleagues [9], we demonstrated that by adopting the RARE-Bestpractices Working Group structure, developing robust CMGs according to the GRADE framework is feasible for FRDA. Adopting the RARE-Bestpractices Working Group framework made it possible to utilize evidence in like conditions, clinical expertise, registry data and feedback from the structured observation forms to make useful recommendations even where evidence in FRDA was low. This process has seen the genesis of 130 new recommendations and 95 best practice statements across 17 broad topic areas.

The addition of new topics was crucial to this iteration. For example, a consistent theme in the development of these guidelines was the frustration of both affected individuals and clinicians alike regarding the experience of someone with FRDA presenting to the emergency room. By most accounts, many emergency room physicians have scarce clinical experience with FRDA and as such may be unsure how to manage issues such as chest pain, trauma or urinary tract infection in the context of FRDA. These CMGs now specifically address this knowledge gap by providing an additional resource to the clinician. Inclusion of such topics reflects the importance of incorporating those who have a lived experience of FRDA and the challenges of navigating the health system, into each step of the development of these guidelines. The inclusion of the lived experience of FRDA provided an important differentiation from the 2014 guidelines and was pivotal in identifying the gaps in those guidelines that required attention. In addition, the inclusion of lay summaries of the recommendations was a significant new initiative. Whilst the target audience of the guidelines is healthcare professionals, provision of a lay summary of the clinical recommendations will assist individuals with FRDA and their families in understanding how best to manage their health and well-being and support shared decision-making with healthcare providers. Lay summaries underwent comprehensive scrutiny by individuals living with FRDA to ensure they address this requirement.

Ensuring the contemporaneous nature and sustainability of recommendations is an important component of guideline development [13]. As new evidence comes to light it is important that recommendations can be modified in real time. The use of the GRADE framework provides not only a robust, transparent structure to the development of the guidelines, but ensures a process of continual updating of the recommendations as new evidence emerges. Moreover, it is anticipated that the guidelines will not only identify the gaps in evidence but also facilitate further studies to address these gaps. Indeed, by the end of this iteration of guideline development, two papers have already arisen from this important process [14, 15].

A further critical component to developing guidelines is dissemination and ensuring the guidelines are openly available and easily accessed by clinicians and others in need of the information. Again, the opinions of clinicians and individuals with a lived experience of FRDA was sought in deciding the best repository and process of dissemination of the CMGs. An important component of dissemination is ensuring transparency, access to the evidence to recommendation tables that underscore the recommendations and the opportunity for future work such as translation into languages other than English. Incorporating suggestions from stakeholders, a dedicated website has been designed to house the CMGs, including chapter content, lay summaries, recommendations, best practice statements and the evidence to recommendation tables. Ongoing review of this process of dissemination will occur via the website.

Finally, it is important to reiterate these CMGs are intended to assist healthcare professionals to make informed treatment decisions about the care of individuals with FRDA. They are not intended as a sole source of guidance in managing issues related to FRDA. Rather, they were designed to assist clinicians by providing an evidence-based framework for decision-making. Ultimately, healthcare professionals must make their own treatment decisions about care on a case-by-case basis, after consultation with individuals living with FRDA, consideration of local or geographical issues including fiscal issues, and using their clinical judgement, knowledge and expertise. Developing guidelines for rare diseases such as FRDA is challenging; however, we believe the blueprint we have provided for this process will be applicable to other clinicians striving, as we are, to provide the best possible clinical care to people who live with rare conditions.

## Supplementary Information

**Additional file 1.** Example of a structured observation form.

**Additional file 2.** Recommendations review survey.

## Data Availability

The datasets used and/or analysed during the current study are available from the corresponding author on reasonable request until such time this manuscript is published. Thereafter the datasets used in this study will be publicly available on a website to be made publically available after publication of this manuscript.

## Abbreviations

EKG: Electrocardiogram
FRDA: Friedreich Ataxia
*FXN*: Frataxin
GAA: Guanine–Adenine–Adenine
LVEF: Left Ventricular Ejection Fraction
NHMRC: National Health and Medical Research Council
NYHA: New York Heart Association
OSA: Obstructive Sleep Apnea
PICO: Patient, Intervention, Comparator, Outcome
RLS: Restless Leg Syndrome
