# Supplementary material for: Clinical management guidelines for Friedreich ataxia: best practice in rare diseases
Source: Orphanet J Rare Dis. 2022 Nov 12;17:415. doi: 10.1186/s13023-022-02568-3 (PMC9652828; doi:10.1186/s13023-022-02568-3)
Supplement: Supplementary file 1 — Additional file 1. Example of a structured observation form. [file 13023_2022_2568_MOESM1_ESM.docx]

**Additional file 2. This is an example of a Structured observation form for the sub-topic of Depression in Friedreich ataxia.**

*This form is to be used in the situation where there is insufficient published evidence and therefore the clinical experience of the expert clinician is sought to inform the Friedreich Ataxia Clinical Management Guidelines.*

| Page 1 of this document outlines a series of questions about the care of individuals with Friedreich ataxia. Please read it, then fill out the items on pages 2 to 3. We are looking not simply for your opinion, but for objective information supported by your observations and/or unpublished data. |
| --- |

**WHAT QUESTIONS DO WE WANT TO ANSWER?**

| 1. **In individuals with Friedreich ataxia with identified depression** |
| --- |
| what is the impact of having:   - Regular counselling to assist in adjusting to transitional events - Review of suicide risk and proactive management - Counselling to manage depression - Pharmacological treatment |
| versus not having these treatments/management strategies, |
| on the following outcomes:   - Depression - Quality of life - Suicide attempts |

| 1. **In individuals with Friedreich ataxia without identified depression** |
| --- |
| What is the impact of having:   - Regular evaluation of risks for developing depression and/or other mental health issues - Regular counselling to assist in adjusting to transitional events - Review of suicide risk and proactive management |
| versus not having these treatments/management strategies, |
| on the following outcomes:   - Depression - Quality of life - Suicide attempts |

**When you think about these questions, please take the perspective of the patient.**

Consider unselected patients, as well as patients:

- With variable access of care (rural versus urban, differing socioeconomic status)
- In children and adults

| Part A The following questions require you to report on your own experience (i.e. your setting and practice, NOT drawing reference to any literature). |
| --- |

Please indicate your country of work:

__________________________________________________________________________________

Describe your role in care of individuals with Friedreich Ataxia.

__________________________________________________________________________________

Describe the setting in which you work.

__________________________________________________________________________________

How many individuals have you cared for with Friedreich ataxia?

__________________________________________________________________________________

Please comment on these patients’ characteristics (e.g. sex, age, ethnicity, access to care, co-morbidities).

__________________________________________________________________________________

| Part B Considering the following management strategies for individuals with Friedreich ataxia **with identified depression**. What is the effect of these strategies on patient-important outcomes? |
| --- |

| **Regular counselling to assist in adjusting to transitional events** | | | | | | |
| --- | --- | --- | --- | --- | --- | --- |
|  | **Large or moderate benefit** | **Small benefit** | **No effect** | **Small harm** | **Large or moderate harm** | **I cannot provide any info on this outcome** |
| Depression |  |  |  |  |  |  |
| Quality of life |  |  |  |  |  |  |
| Suicide attempts |  |  |  |  |  |  |

| **Review of suicide risk and proactive management** | | | | | | |
| --- | --- | --- | --- | --- | --- | --- |
|  | **Large or moderate benefit** | **Small benefit** | **No effect** | **Small harm** | **Large or moderate harm** | **I cannot provide any info on this outcome** |
| Depression |  |  |  |  |  |  |
| Quality of life |  |  |  |  |  |  |
| Suicide attempts |  |  |  |  |  |  |

| **Counselling to manage depression** | | | | | | |
| --- | --- | --- | --- | --- | --- | --- |
|  | **Large or moderate benefit** | **Small benefit** | **No effect** | **Small harm** | **Large or moderate harm** | **I cannot provide any info on this outcome** |
| Depression |  |  |  |  |  |  |
| Quality of life |  |  |  |  |  |  |
| Suicide attempts |  |  |  |  |  |  |

| **Pharmacological treatment** | | | | | | |
| --- | --- | --- | --- | --- | --- | --- |
|  | **Large or moderate benefit** | **Small benefit** | **No effect** | **Small harm** | **Large or moderate harm** | **I cannot provide any info on this outcome** |
| Depression |  |  |  |  |  |  |
| Quality of life |  |  |  |  |  |  |
| Suicide attempts |  |  |  |  |  |  |

| Part C Considering the following management strategies for individuals with Friedreich ataxia **without identified depression**. What is the effect of these strategies on patient-important outcomes? |
| --- |

| **Regular evaluation of risks for developing depression and/or other mental health issues** | | | | | | |
| --- | --- | --- | --- | --- | --- | --- |
|  | **Large or moderate benefit** | **Small benefit** | **No effect** | **Small harm** | **Large or moderate harm** | **I cannot provide any info on this outcome** |
| Depression |  |  |  |  |  |  |
| Quality of life |  |  |  |  |  |  |
| Suicide attempts |  |  |  |  |  |  |

| **Regular counselling to assist in adjusting to transitional events** | | | | | | |
| --- | --- | --- | --- | --- | --- | --- |
|  | **Large or moderate benefit** | **Small benefit** | **No effect** | **Small harm** | **Large or moderate harm** | **I cannot provide any info on this outcome** |
| Depression |  |  |  |  |  |  |
| Quality of life |  |  |  |  |  |  |
| Suicide attempts |  |  |  |  |  |  |

| **Review of suicide risk and proactive management** | | | | | | |
| --- | --- | --- | --- | --- | --- | --- |
|  | **Large or moderate benefit** | **Small benefit** | **No effect** | **Small harm** | **Large or moderate harm** | **I cannot provide any info on this outcome** |
| Depression |  |  |  |  |  |  |
| Quality of life |  |  |  |  |  |  |
| Suicide attempts |  |  |  |  |  |  |
