# Supplementary material for: Clinical management guidelines for Friedreich ataxia: best practice in rare diseases
Source: Orphanet J Rare Dis. 2022 Nov 12;17:415. doi: 10.1186/s13023-022-02568-3 (PMC9652828; doi:10.1186/s13023-022-02568-3)
Supplement: Supplementary file 2 — Additional file 2. Recommendations review survey. [file 13023_2022_2568_MOESM2_ESM.docx]

**Additional file 2: Panel endorsement of Clinical Management Guidelines for Friedreich ataxia.**

**Chapter number and chapter title:**

**If relevant, number and subtitle of topic:**

**Name of reviewer:**

**Date:**

**Questions.**

1. The overall recommendation section (including recommendation, justification and sub-group if appropriate) chapter is clearly written in an understandable form.

Yes

Partly

No

Please comment on your answer:

|  |
| --- |

1. The background to the recommendations as well as the cohort to whom the guideline is meant to apply are clearly described.

Yes

Partly

No

Please comment on your answer:

|  |
| --- |

1. The recommendations are unambiguous and presented along with the grade (type) of recommendation (as indicated above) and supporting evidence (justification). Please indicate in comments if you think it is helpful to include the type of recommendation according to GRADE (as currently presented in brackets after the recommendation i.e., *We suggest using insulin alone rather than insulin and other glucose-lowering therapy as the primary treatment for most children (< 18 years) with FRDA-related diabetes mellitus (conditional recommendation for the intervention based on very low certainty of evidence of effect).*

Yes

Partly

No

Please comment on your answer:

|  |
| --- |

1. The different options for the management of the condition are clearly presented considering the health benefits, side effects and risks.

Yes

Partly

No

Please comment on your answer:

|  |
| --- |

1. Please indicate if from your perspective the recommendation(s)is (are) suitable for endorsement as a guideline

Yes

No

**Comments**

|  |
| --- |
